# FSRT vs. SRS in Brain Metastases—Differences in Local Control and Radiation Necrosis—A Volumetric Study

**DOI:** 10.3389/fonc.2020.559193

**Published:** 2020-09-30

**Authors:** Florian Putz, Thomas Weissmann, Dominik Oft, Manuel Alexander Schmidt, Johannes Roesch, Hadi Siavooshhaghighi, Irina Filimonova, Charlotte Schmitter, Veit Mengling, Christoph Bert, Benjamin Frey, Sebastian Lettmaier, Luitpold Valentin Distel, Sabine Semrau, Rainer Fietkau

**Affiliations:** ^1^Department of Radiation Oncology, Universitätsklinikum Erlangen, Friedrich-Alexander-Universität Erlangen-Nürnberg, Erlangen, Germany; ^2^Department of Neuroradiology, Universitätsklinikum Erlangen, Friedrich-Alexander-Universität Erlangen-Nürnberg, Erlangen, Germany

**Keywords:** brain metastases, stereotactic radiotherapy (SRT), stereotactic radiosurgery (SRS), fractionated stereotactic radiotherapy, volumetric analysis, segmentation, radiobiological aspects

## Abstract

**Background:** While the role of stereotactic radiotherapy for brain metastases is increasing, evidence on the comparative efficacy and safety of fractionated stereotactic radiotherapy (FSRT) and single-session radiosurgery (SRS) is scarce.

**Methods:** Longitudinal volumetric analysis was performed in a consecutive cohort of 120 patients and 190 brain metastases (>0.065 cm^3^ in volume / > ~5 mm in diameter) treated exclusively with FSRT (*n* = 98) and SRS (*n* = 92), respectively. A total of 972 tumor segmentations was used, averaging 5.1 time points per metastasis. Progression was defined using a volumetric extension of the RANO-BM criteria. Local control and radionecrosis were compared for lesions treated with FSRT and SRS, respectively.

**Results:** Metastases treated with FSRT were significantly larger at baseline (mean, 4.66 vs. 0.40 cm^3^, *p* < 0.001). Biologically effective dose (BED) for metastases (α/β = 12, linear-quadratic-cubic model) was significantly associated with local control, whereas BED for normal brain (α/β = 2, linear-quadratic model) was significantly associated with radionecrosis. Median time to local progression was 22.9 months in the FSRT group compared to 14.5 months in the SRS group (*p* = 0.022). Overall radionecrosis rate at 12 months was 3.4% for FSRT and 14.8% for SRS (*p* = 0.010). Radionecrosis °IV requiring resection with histologic proof of radiation necrosis also was significantly reduced in the FSRT group (FSRT 0.0% vs. SRS 3.9%, *p* = 0.041). In multivariate analysis, FSRT was associated with reduced risk of progression (HR 0.47, *p* = 0.015) and reduced risk of radionecrosis (HR 0.18, *p* = 0.045).

**Conclusions:** This volumetric study provides initial evidence that the improvements in therapeutic ratio expected for FSRT in larger brain metastases, might equally extend into the domain of smaller metastases, traditionally less considered for fractionated treatment. FSRT might constitute an important tool to further increase local control and reduce radionecrosis risk in stereotactic radiotherapy for brain metastases, that should be assessed in randomized intervention trials.

## Introduction

Stereotactic radiotherapy (SRT) is one of the most important treatments for brain metastases, an increasingly common disease entity that occurs in up to 40% of patients with cancer (1). Advances in systemic treatments like immunotherapies and targeted agents increasingly enable extracranial long-term control and consequently heighten the significance of effective and safe intracranial radiotherapy (2, 3). At the same time, radiooncologists are increasingly reserved about using Whole-brain radiotherapy (WBRT) in the face of potential neurocognitive side effects (4, 5). Foregoing adjuvant WBRT, however, not only increases the risk of distant brain metastases, but has also profound impact on local control of stereotactically irradiated brain metastases: In three randomized controlled trials that evaluated the role of adjuvant WBRT in patients treated with radiosurgery, local control consistently diminished from around 90% to only 70% at 12 months in patients without WBRT (4, 6, 7). If long-term survival depends on lasting intra-cranial control, local efficacy of SRT in brain metastases needs to improve without increasing toxicity, especially when considering that patients frequently suffer from multiple lesions.

Fractionated stereotactic radiotherapy (FSRT) may constitute an important option to increase the therapeutic ratio in comparison to single-session radiosurgery (SRS) in patients with brain metastases. In addition to being a treatment of equally high spatial precision, FSRT could leverage fundamental radiobiological differences between brain metastases and surrounding normal brain tissue (8). As current radiobiologic understanding suggests that brain metastases have a very high α/β ratio of around 12, whereas surrounding normal brain tissue is characterized by a low α/β of 2-3, dose fractionation should—in theory—be able to optimize local control while avoiding increased risk for radionecrosis (8–10). On the other hand, large single doses in excess of 8 Gy have been shown to cause endothelial cell apoptosis via the acid sphingomyelinase pathway, which has been discussed to enhance the effect of SRS in comparison to fractionated treatment (11, 12). However, while SRS has been the first and foremost modality investigated in prospective studies, evidence on FSRT is still scarce and especially comparative analyses between FSRT and SRS are mostly lacking (13).

In this study, we explore potential differences in local control and radionecrosis between metastases treated with SRS and FSRT using a longitudinal volumetric analysis based on 972 tumor segmentations in 190 metastases to enable an accurate assessment of even small lesions and to account for any baseline volume differences. Volumetric criteria for progression have been defined objectively by deriving them from the current unidimensional RANO-BM criteria (14).

## Materials and Methods

### Patient Population

We identified all patients who received stereotactic radiotherapy (SRT) for intracranial metastases at our institution between January of 2003 and April of 2015. This retrospective analysis was in accordance with the ethical standards of the institutional research committee and with the 1964 Helsinki declaration and its later amendments. Based on local legislation (BayKrG Art. 27), consent for study inclusion was available in all patients. From this group of 566 patients, patients were selected based on the following inclusion criteria: (1) stereotactic radiotherapy for intraparenchymal brain metastases from a solid cancer, (2) no prior SRT and no prior resection of the metastasis to be analyzed, (3) availability of contrast-enhanced T1-Mprage sequences with ≤1 mm slice thickness at baseline and at least once during follow-up. Four hundred and nineteen brain metastases in 189 patients fulfilled these criteria. Analyses were conducted at the level of individual metastases. For the present study, all metastases from this cohort were used that had a minimum baseline volume of >0.065 cm^3^ (corresponding to a minimum diameter of >5 mm for a spherical lesion consistent with current RANO-BM recommendations to be considered measurable) and had not received concurrent WBRT. Metastases, in which WBRT was delivered ≥8 weeks (56 days) prior to SRT were entered into the analysis with former WBRT being included as variable in multivariate analyses. In total, 190 metastases fulfilled these criteria and were used in the present study.

### Radiation Therapy

Patients received single-session radiosurgery (SRS) or fractionated stereotactic radiotherapy (FSRT) with a linear accelerator based Novalis® or Novalis-Tx® system (BrainLAB, Feldkirchen, Germany). Patients were assigned to FSRT instead of SRS according to tumor size and the presence of adjacent Organs at Risk. Instead of a formal diameter or volume threshold for selecting lesions for SRS and FSRT, respectively, metastases were selected for FSRT, if SRS treatment was expected to result in a V10Gy for normal brain of over 10 cm^3^, which is a well-recognized threshold for increased risk of radiation necrosis as established by Blonigen et al. (15). Patients were immobilized in an individually manufactured thermoplastic head mask attached to a stereotactic base frame (BrainLAB, Feldkirchen, Germany). Treatment planning was performed using Iplan (BrainLAB, Feldkirchen, Germany) (16, 17). Patients received a dedicated planning CT, which was rigidly co-registered with the baseline MRI using the Iplan software. The gross target volume (GTV) was delineated in the contrast-enhanced T1-Mprage sequence of the baseline MRI study. Planning target volume (PTV) was defined as GTV with an additional margin of 1–2 mm. Dose was prescribed to the encompassing 80%-isodose. During treatment, daily stereoscopic X-ray imaging (ExacTrac®) was used for setup verification and repositioning. For SRS stereoscopic X-ray imaging was repeated after every couch rotation. As established by Wiggenraad et al., biologically effective dose (BED) for brain metastases was calculated based on an α/β ratio of 12 according to the LQC model (BED_12−LQC_) (10, 18):

$$\begin{array}{l} \text{BE}{\text{D}}_{12-\text{LQC}}=nd\left[1+\frac{d}{\left(\frac{\alpha }{\beta }\right)}-\frac{d^{2}}{\left(\frac{\alpha }{\gamma }\right)}\right] \end{array}$$

With *n* being the number of fractions and *d* being the dose per fraction, α*/*β was assumed to be 12 Gy and α/γ 648 Gy^2^ (10, 18). In addition, to model BED for normal brain tissue, BED_2−LQ_ was calculated based on an α/β of 2 according to the conventional linear-quadratic model. Median SRS dose was 18.0 Gy. FSRT was delivered daily excluding weekends, median FSRT single dose was 4.0 Gy, median fraction number was 10 and median total FSRT dose was 40.0 Gy (Table 1).

**Table 1:** Characteristics of treated brain metastases (*N* = 190).

**Metastasis characteristic**	**SRS (*N* = 92)**	**FSRT (*N* = 98)**	***p* for comparison**
**Pretreatment metastasis volume, cm**3			*p* < 0.001^#^
Median (IQR)	0.23 (0.12–0.50)	1.42 (0.34–4.41)	
Mean (range)	0.40 (0.07–2.38)	4.66 (0.07–61.98)	
**Pretreatment metastasis diameter, cm**			*p* < 0.001^#^
Median (IQR)	1.0 (0.8–1.3)	1.8 (1.1–2.7)	
Mean (range)	1.1 (0.6–2.1)	2.1 (0.6–6.1)	
**Histology**, ***n*** **(%)**			*p* = 0.005^§^
Melanoma	51 (55.4%)	32 (32.7%)	
Lung	9 (9.8%)	17 (17.3%)	
Breast	4 (4.3%)	16 (16.3%)	
Renal	15 (16.3%)	15 (15.3%)	
Other	13 (14.1%)	18 (18.4%)	
**Single Dose, Gy**			*p* < 0.001^#^
Median (IQR)	20.0 (18.0–20.0)	4.0 (4.0–6.0)	
Mean (range)	19.3 (16.0–20.0)	4.5 (2.0–7.0)	
**Total Dose, Gy**			*p* < 0.001^#^
Median (IQR)		40.0 (35.0–40.0)	
Mean (range)		39.1 (28.0–54.0)	
**BED**_**12-LQC**_**, Gy**			*p* < 0.001^#^
Median (IQR)	41.0 (36.0–41.0)	52.4 (52.4–52.8)	
Mean (range)	39.2 (31.0–41.0)	52.2 (36.6–63.0)	
**BED**_**2-LQ**_**, Gy**			*p* < 0.001^#^
Median (IQR)	220.0 (180.0–220.0)	120.0 (120.0–144.0)	
Mean (range)	205.8 (144.0–220.0)	125.4 (72.0–157.5)	
**Former Whole-brain radiotherapy**, ***n*** **(%)**			*p* = 0.351^§^
Yes	19 (20.7%)	15 (15.3%)	
No	73 (79.3%)	83 (84.7%)	

*IQR, interquartile range; BED_12−LQC_, Biologically effective dose for an alpha/beta ratio of 12, linear-quadratic-cubic model; BED_2−LQ_, Biologically effective dose for an alpha/beta ratio of 2, linear-quadratic model*.

# *T-Test*.

§ *Fisher's exact text*.

### Follow-Up and Imaging

Images were collected on different Siemens 1.5 Tesla MRI scanners (Magnetom Aera or Magnetom Avanto) at our institution. All analyzed images consisted of 160 or 192 contiguous, sagittal, or transverse planes of 3-dimensional T1-weighted magnetization-prepared rapid gradient-echo images with 1 × 1 × 1 mm isotropic resolution (repetition time [TR] = 1,900 ms, echo time [TE] = 3.02 ms, inversion time [TI] = 1,100 ms, matrix = 256 × 265, field of view [FoV] = 250, flip angle = 15 degrees or TR = 2,200 ms, TE = 2.67 ms, TI = 900 ms, matrix = 256 × 246, FoV = 250, flip angle = 8 degrees) after intravenous application of 0.2 mL/kg Dotarem (Guerbet) or 0.1 mL/kg Gadovist (Bayer), respectively.

Patients received MRI at baseline (median of 8 days prior to radiotherapy) and routinely at 6 weeks after stereotactic radiotherapy (SRT) and every 3 months thereafter. However, due to the retrospective nature of the study patients received MRI at slightly different points in time after SRT.

### Volumetric Analysis

In 190 brain metastases, 972 time points / MRI studies were available at baseline or following SRT and segmented longitudinally corresponding to a mean of 5.1 segmentations per metastasis (median of 4 [range 2–30]), which means that lesions were measured on an average of 5.1 separate MRI studies conducted at different points in time during follow-up and including one baseline measurement before treatment per lesion. Segmentation was performed using the open-source software 3D Slicer (version 4.5.0) (19). 3D Slicer is supported by the National Institutes of Health (NIH) (20) and offers different modules for segmentation, volume statistics and image coregistration. A custom-developed module was used that utilizes the built-in modules but accelerates the segmentation process by automating steps that do not require user interaction (21). Segmentation was performed semi-automatically using the VTK Fast Growcut method (22) as semiautomatic segmentation methods have been shown to decrease inter- and intra-observer variabilities (23, 24) and are much more time-efficient than manual delineation (25). Following a first semi-automatic segmentation step all segmentations were reviewed and corrected manually on a slice-by-slice basis using the editor module in 3D Slicer. All segmentations were reviewed, corrected and validated by an experienced radiation oncologist who is further specialized in neuro-oncology, segmentation, and imaging.

### Volumetric Extension of the RANO-BM Criteria for the Assessment of Progression Following SRT

**Figure 1:**
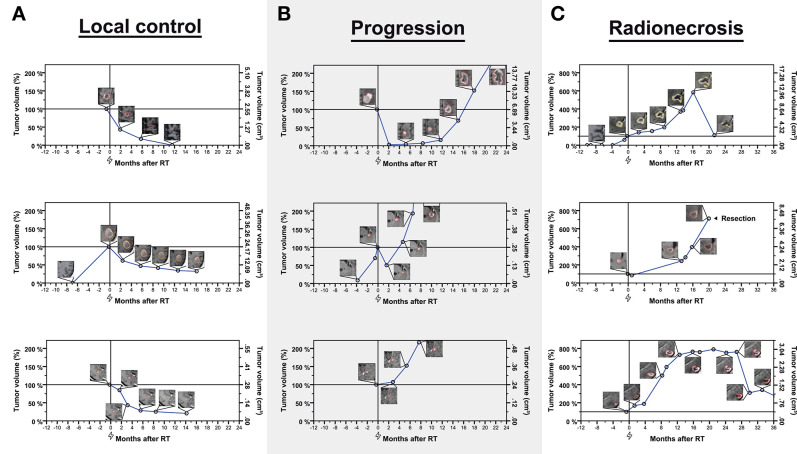
Examples of longitudinal volumetry in nine brain metastases classified as controlled (**A**—left column), experiencing progression (**B**—middle columns), or radionecrosis (**C**—right column). Tumor volumes are expressed relative to baseline volume (left y-axis) over time. The right y-axis shows the absolute metastasis volume in cm^3^. The flash symbol indicates the time of radiotherapy (0 months−100% relative tumor volume). Inlay images show segmented metastases for different measurements. In total, 972 time points in 190 brain metastases were used in this study (average of 5.1 per metastasis).

We adopted the basic concept from the RANO-BM guideline to derive volumetric criteria from the established unidimensional recommendations using spherical geometry. In this regard, the RANO-BM guideline recommends defining volumetric partial response as ≥65% reduction in volume corresponding to a spherical lesion shrinking by ≥30% in diameter, which is the current unidimensional definition of partial response (14). Following this principle, progression was defined as ≥72.8% increase in volume in the present study relative to nadir/baseline, which corresponds to a ≥20% increase in diameter of a perfect sphere (i.e., the unidimensional RANO-BM criteria for progression) (Figure 1). In addition, as the RANO-BM guideline recommends to consider small brain metastases between 5 and 10 mm in diameter as unchanged unless the longest diameter changes by at least 3 mm, an additional absolute increase in volume of at least 0.2 cm^3^ was required for the definition of progression in the present study. This corresponds to the absolute volume increase of a 5 mm sphere growing by additional 3 mm in diameter. In addition, as SRT is a localized therapy, change in distant lesions, corticosteroid use or clinical status were not considered in the definition of progression in the present study.

Lesions that fulfilled these volumetric criteria for progression but showed spontaneous regression during subsequent imaging follow-up were classified as radionecrosis instead of progression. Spontaneous regression was defined as regression to baseline/nadir volume or volumetric partial response as per the RANO-BM recommendation (i.e., ≥65% reduction in volume) without any additional treatment (local or systemic) that could explain tumor shrinkage. According to RTOG 9005, radiation necrosis requiring resection was classified grade IV and metastases were classified as progression, radionecrosis or both based on histology (Figure 1) (26).

### Conventional Unidimensional Assessment of Metastases Diameters

In addition to the volumetric assessment described above, we measured metastases diameters in all lesions and assessed progression according to current unidimensional RANO-BM criteria with an increase of ≥20.0% in diameter being required for the definition of local progression. In addition, a minimum absolute increase of 3 mm in diameter was required for lesions smaller than 10 mm as per the current RANO-BM recommendations (14). Similar to serial volumetric measurements, radionecrosis also was assessed using sequential unidimensional measurements. Lesions that fulfilled the unidimensional criteria for progression but showed spontaneous regression during serial imaging follow-up were classified as radionecrosis instead of progression. Spontaneous regression was defined as regression of tumor diameter back to nadir/baseline or partial response according to RANO-BM criteria (i.e., ≥30% reduction in diameter) in the absence of any additional treatment (local or systemic) that could explain tumor shrinkage.

### Statistical Analysis

Time-to-event outcomes were calculated from the start of SRT to the date of first MRI demonstrating volumetric progression according to the criteria described above and evaluated using the Kaplan-Meier estimator and the Log-rank test. In case of resection without prior volumetric progression on MRI, time-to-event outcomes were calculated to the day of the intervention. Data were censored at the date of the last imaging assessment. Covariates were included in multivariate Cox's models based on biologic considerations. *P* < 0.05 were considered statistically significant. All statistical analyses were performed using IBM SPSS 21.

## Results

One hundred and ninety brain metastases in 120 unique patients were subjected to longitudinal volumetric analysis (Figure 1). In total, 972 whole-tumor segmentations were available after stereotactic radiotherapy and at baseline with a mean of 5.1 segmentations per metastasis (median of 4 [range 2–30]), which means that lesions were measured on an average of 5.1 separate MRI studies conducted at different points in time during follow-up and including one baseline measurement before treatment per lesion. Median imaging follow-up was 7.4 months (95%CI, 5.0–9.7 months). Median time from radiotherapy to death was 10.4 months (95%CI, 8.7–12.1 months). There was no significant difference in overall survival between the FSRT and SRS groups, respectively (logrank *p* = 0.158) with overall survival being numerically lower in the SRS arm (median overall survival, 7.5 vs. 11.5 months for SRS vs. FSRT, respectively).

**Figure 2:**
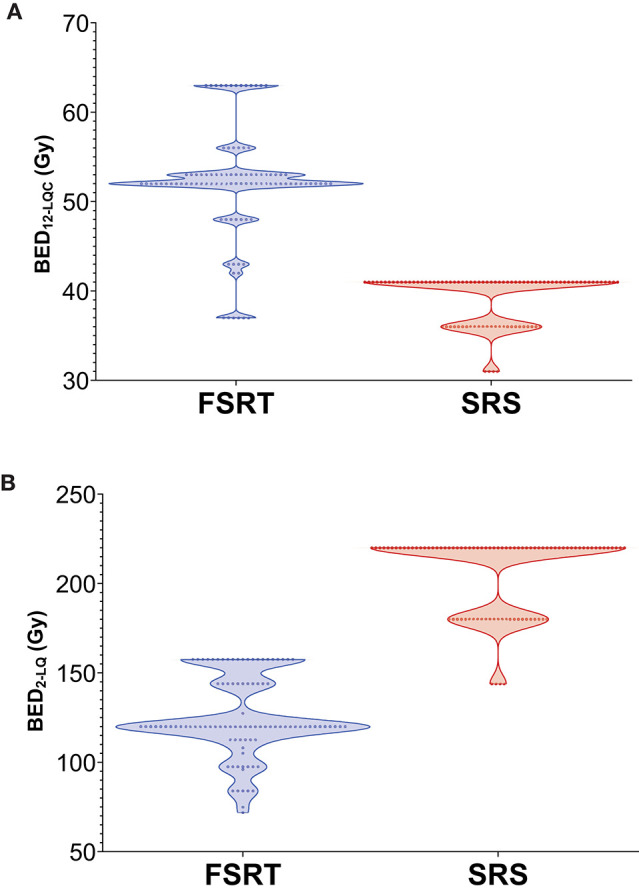
Violin Plot showing the distribution of BED_12−LQC_ **(A)** and BED_2−LQ_ **(B)** for metastases treated with FSRT and SRS, respectively. BED_12−LQC_ = biologically effective dose for an α/β ratio of 12, linear-quadratic-cubic model. BED_2−LQ_ = biologically effective dose for an α/β ratio of 2, linear-quadratic model.

Ninety-two metastases (48.4%) had been treated with stereotactic radiosurgery (SRS), while 98 tumors (51.6%) had received fractionated stereotactic radiotherapy (FSRT). Metastases treated with FSRT were highly significantly larger than those treated with SRS (Mean volume at radiotherapy, 4.66 vs. 0.40 cm^3^, *p* < 0.001). In addition, the distribution of primary tumors was different between metastases treated with FSRT and SRS (*p* = 0.005), including more melanoma brain metastases in the SRS group. When calculating biologically effective doses (BED) for the SRS and FSRT treatment groups, the BED for an α/β ratio of 12 according to the linear-quadratic-cubic model (BED_12−LQC_) was highly significantly larger in the FSRT arm (mean, 52.2 vs. 39.2 Gy) whereas the mean BED for an α/β ratio of 2 according to the linear-quadratic model (BED_2−LQ_) was highly significantly smaller in metastases treated with FSRT compared to SRS (125.4 vs. 205.8 Gy) (Table 1, Figure 2).

Progression and radiation necrosis following stereotactic radiotherapy were defined according to a volumetric extension of the RANO-BM criteria (see methods section) or according to histology, if metastases were resected after radiotherapy. In addition to the volumetric assessment, all metastases were evaluated conventionally using diameter measurements according to current unidimensional RANO-BM criteria.

### Volumetric Control in Metastases Treated With FSRT Compared to SRS

There was a total of 54 progression events following radiotherapy, of which 52 were based on volumetric progression on follow-up MRI and 2 were based on histologic findings after resection alone.

**Figure 3:**
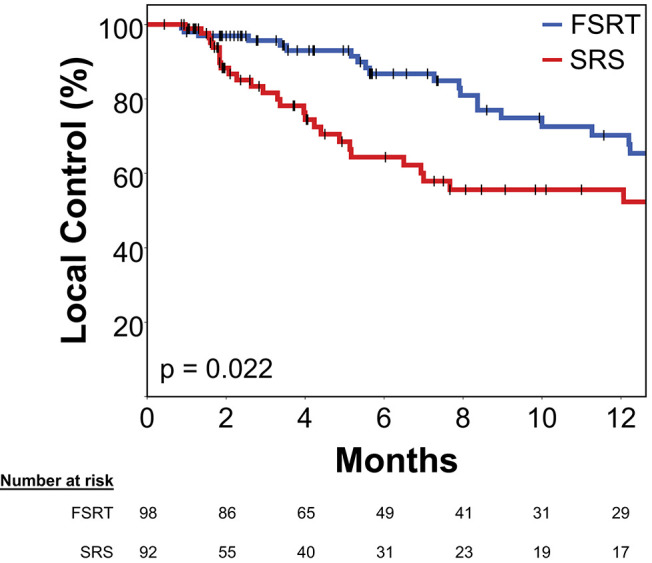
Local control in 92 brain metastases treated with SRS (red) and 98 brain metastases treated with FSRT (blue). Log-rank *p* = 0.022. Progression was defined volumetrically or as tissue diagnosis showing viable tumor after radiotherapy (see Methods section for details). Vertical lines represent censored cases. SRS, Stereotactic radiosurgery; FSRT, Fractionated stereotactic radiotherapy.

Median time to local progression was 22.9 months in the FSRT group compared to 14.5 months in the SRS group (Log-rank *p* = 0.022). One-year local control was 70.2% in metastases treated with FSRT and 55.6% for lesions treated with SRS (Figure 3).

In multivariate Cox's regression analysis, metastases treated with FSRT had a significantly smaller risk of progressing than those treated with SRS (HR 0.47, *p* = 0.015). Primary tumor histology had a significant impact in multivariate analysis with melanoma histology being an adverse factor for local control. In addition, former WBRT was associated with significantly higher risk of local progression (HR 4.24, *p* = 0.001), whereas pretreatment metastasis volume (HR 1.01 per cm^3^, *p* = 0.608) had no significant effect in multivariate analysis (Table 2).

**Table 2:** Prognostic factors for local control on univariate and multivariate Cox's regression analysis (*N* = 190).

**Parameter**	**Univariate**		**Multivariate**	
	**HR (95% CI)**	***p*-value**	**HR (95% CI)**	***p*-value**
FSRT vs. SRS	0.54 (0.31–0.92)	**0.024**	0.47 (0.25–0.87)	**0.015**
Former Whole-brain radiotherapy	2.15 (1.13–4.11)	**0.021**	4.24 (1.75–10.26)	**0.001**
Primary tumor histology		**0.011**		**0.003**
Lung vs. Melanoma	0.51 (0.21–1.21)	0.127	0.38 (0.15–0.98)	**0.044**
Breast vs. Melanoma	0.68 (0.30–1.54)	0.350	0.37 (0.12–1.08)	0.069
Renal vs. Melanoma	0.23 (0.08–0.65)	**0.006**	0.23 (0.08–0.66)	**0.006**
Other vs. Melanoma	0.28 (0.10–0.80)	**0.017**	0.21 (0.07–0.62)	**0.005**
Pretreatment metastasis volume, cm^3^	0.99 (0.94–1.03)	0.54	1.01 (0.97–1.06)	0.608
BED_12−LQC_, Gy	0.94 (0.90–0.98)	**0.002**	Not included	
BED_2−LQ_, Gy	1.00 (1.00–1.01)	0.247	Not included	

*HR, Hazard ratio; WBRT, Whole-brain radiotherapy; FSRT, Fractionated stereotactic radiotherapy; SRS, Stereotactic radiosurgery; BED_*12*−*LQC*_, Biologically effective dose for an alpha/beta ratio of 12, linear-quadratic-cubic model; BED_*2*−*LQ*_, Biologically effective dose for an alpha/beta ratio of 2, linear-quadratic model*.

*BED_*12*−*LQC*_ was not included in multivariate analysis because it was highly correlated with the type of stereotactic radiotherapy (FSRT vs. SRS)*.

*Bold values indicate statistical significance*.

Higher BED_12−LQC_ was strongly associated with improved local control in univariate analysis for local control (HR 0.94 per Gy, *p* = 0.002) but was not included in the multivariate model as it was highly correlated to the type of stereotactic radiotherapy (FSRT vs. SRS).

To further address differences in the proportion of melanoma brain metastases between the FSRT and the SRS group, we separately evaluated local control in metastases with and without melanoma histology. Interestingly, when comparing local control between FSRT and SRS exclusively in metastases with melanoma histology (*n* = 83), local control still was profoundly improved in the FSRT arm (12-months local control, 59.8 vs. 46.6%, *p* = 0.069) and when limiting the analysis to non-melanoma brain metastases only (*n* = 107), local control also was improved in the FSRT group (12-months local control, 76.0 vs. 67.8%, *p* = 0.871) with differences being less pronounced, however. To additionally assess the impact of tumor size on the comparative effectiveness of FSRT and SRS, we separately compared local control for lesions below and above 1 cm in diameter. For lesions <1.0 cm in diameter, local control at 12 months was 68.6% for FSRT-treated lesions (*N* = 14), while 12-months local control was 65.4% in the SRS group (*N* = 43, logrank *p* = 0.855). In lesions ≥1.0 cm in diameter, differences were more pronounced and 12-months local control was 71.0% in FSRT-treated metastases (*N* = 84), while it was only 47.7% in lesions treated with SRS (*N* = 49, logrank *p* = 0.003).

### Rate of Radionecrosis for FSRT vs. SRS

In total, two radionecrosis events (grade I-IV) were observed in the FSRT group, whereas 8 events occurred in the SRS group.

**Figure 4:**
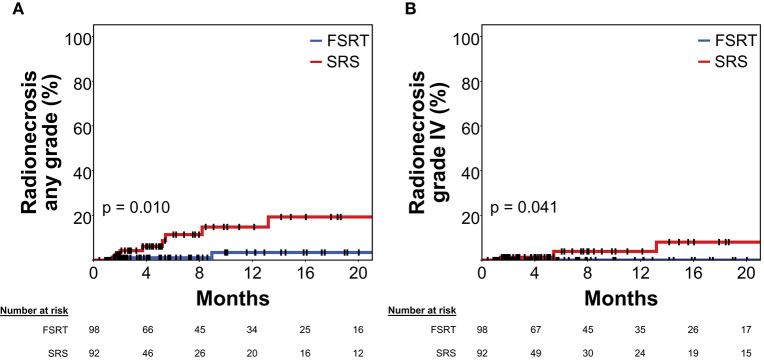
Rate of radionecrosis of any grade **(A)** and grade IV radionecrosis **(B)** in 92 brain metastases treated with SRS (red) and 98 brain metastases treated with FSRT (blue). Log-rank *p* = 0.010 and *p* = 0.041, respectively. Radionecrosis was defined volumetrically or as tissue diagnosis showing radionecrosis after radiotherapy (see Methods section for details). As radionecrosis grade IV is defined as requiring surgical intervention, all events shown in **(B)** had histologic proof of radionecrosis. Vertical lines represent censored cases. SRS, Stereotactic radiosurgery; FSRT, Fractionated stereotactic radiotherapy.

At 12 months post-RT, radionecrosis rate (grade I–IV) was 3.4% in metastases treated with FSRT compared to 14.8% in tumors treated with SRS (Figure 4A, Log-rank *p* = 0.010).

In multivariate Cox's regression analysis, FSRT continued to be associated with significantly reduced risk for radiation necrosis (HR 0.18, *p* = 0.045), when included among pretreatment tumor volume and former WBRT. BED_2−LQ_ was significantly associated with increased risk for radionecrosis in univariate Cox's regression (HR 1.02 per Gy, *p* = 0.035) but was not included in the multivariate model, as it was highly correlated to the type of stereotactic radiotherapy (FSRT vs. SRS) (Table 3).

**Table 3:** Predictive factors for radionecrosis on univariate and multivariate Cox's regression analysis (*N* = 190).

**Parameter**	**Univariate**		**Multivariate**	
	**HR (95% CI)**	***p*-value**	**HR (95% CI)**	***p*-value**
FSRT vs. SRS	0.17 (0.04–0.78)	**0.023**	0.18 (0.03–0.96)	**0.045**
Past Whole-brain radiotherapy	1.52 (0.32–7.16)	0.596	1.85 (0.38–8.99)	0.449
Pretreatment metastasis volume, cm^3^	0.77 (0.45–1.31)	0.338	0.95 (0.69–1.32)	0.755
Checkpoint inhibitor therapy	0.93 (0.12–7.4)	0.944	0.71 (0.09–5.85)	0.746
BED_2−LQ_, Gy	1.02 (1.00–1.03)	**0.035**	Not included	
BED_12−LQC_, Gy	0.92 (0.84–1.02)	0.116	Not included	

*HR, Hazard ratio; WBRT, Whole-brain radiotherapy; FSRT, Fractionated stereotactic radiotherapy; SRS, Stereotactic radiosurgery; BED_2−LQ_, Biologically effective dose for an alpha/beta ratio of 2, linear-quadratic model; BED_12−LQC_, Biologically effective dose for an alpha/beta ratio of 12, linear-quadratic-cubic model*.

*BED_2−LQ_ was not included in multivariate analysis because it was highly correlated with the type of stereotactic radiotherapy (FSRT vs. SRS)*.

*Bold values indicate statistical significance*.

Only in 13 out of 190 lesions, immunotherapy using PD-1 or CTLA-4 inhibitors was delivered at any time prior to or after radiotherapy. Probably because of the low frequency of checkpoint inhibitor treatment in the present series, immunotherapy was not significantly associated with the risk of developing radionecrosis in univariate or multivariate analysis (multivariate HR = 0.71, *p* = 0.746) (Table 3). Lastly, analysis was limited to grade IV radionecrosis alone that per definition requires resection and thus had tissue diagnosis of radionecrosis in all cases. At 12 months post-RT, grade IV radionecrosis rate was 0.0% in metastases treated with FSRT compared to 3.9% in tumors treated with SRS (Figure 4B, Log-rank *p* = 0.041).

To additionally assess the impact of tumor size on the rate of radionecrosis following FSRT and SRS, respectively we separately compared radionecrosis for lesions below and above 1 cm in diameter. For lesions <1.0 cm in diameter, 12-months radionecrosis rate was 0% in FSRT-treated metastases (*N* = 14), while it was 9.6% for SRS-treated lesions (*N* = 43, logrank *p* = 0.257).

For metastases ≥1.0 cm, differences were more pronounced and 12-months radionecrosis rate was 3.9% in the FSRT group (*N* = 84), while it reached 21.2% in metastases >1.0 cm treated with SRS (*N* = 49, logrank *p* = 0.006).

### Conventional Assessment of Progression Using Unidimensional RANO-BM Criteria

In addition to the volumetric assessment, all metastases were evaluated conventionally using diameter measurements according to current unidimensional RANO-BM criteria. Sixty-two progression events were observed with unidimensional assessment compared to 54 progression events using volumetric measurements. Median local control for unidimensional assessment was 22.9 months in the FSRT and 8.2 in the SRS group (logrank *p* = 0.001). For radionecrosis, a total of 13 events were observed using unidimensional assessment as compared to 10 using volumetric measurements. Using serial unidimensional measurements, 12-month radionecrosis rate was 3.7% in the FSRT and 17.3% in the SRS group (logrank *p* = 0.036).

## Discussion

Single-fraction stereotactic radiosurgery (SRS) according to RTOG 9005 continues to be the international gold standard for stereotactic radiotherapy (SRT) of brain metastases. RTOG 9005 was a dose-finding study by the Radiation Therapy Oncology Group (RTOG), which investigated increasing SRS doses for the treatment of brain metastases (26). A crucial finding of the study was that radionecrosis profoundly increased with tumor diameter in the absence of dose reduction. The recommendation derived from this study, therefore, has been to irradiate brain metastases ≤2 cm in diameter with a single dose of 24 Gy and larger metastases with reduced doses of 18 Gy (2–3 cm) and 15 Gy (3–4 cm), respectively (26). As SRS is the first and foremost modality investigated in prospective studies on the treatment of brain metastases to date, it is an integral part of national and international treatment guidelines (13, 27). Fractionated stereotactic radiotherapy (FSRT) on the other hand is much less well-studied and therefore only plays a minor role in official recommendations with the main use of fractionation being seen in larger lesions of >3 cm diameter, where single-session treatment is obviously limited by toxicity or the need for dose reduction (13, 28, 29).

While evidence on the comparative efficacy and safety of FSRT and SRS is scarce and prospective data does not exist, some well-designed retrospective studies have been undertaken. Minniti et al. conducted a retrospective comparison of FSRT and SRS in 289 patients and 343 brain metastases of >2 cm diameter (30). SRS generally was performed following the RTOG 9005 recommendations and FSRT consisted of 3 × 9 Gy fractions. Progression had been defined as increase in tumor diameter in at least two consecutive MRIs, whereas the distinction with radionecrosis was made using MRI perfusion and F-DOPA PET. In this cohort of larger metastases (>2 cm), the authors found a significant increase in local control for FSRT of 91% compared to 77% at 12 months (*p* = 0.01) for SRS. In addition, radiation necrosis decreased from 18 to 9%, if metastases had been treated with FSRT (*p* = 0.01) (30).

In a recent study of 105 metastases sized 2.5–3 cm, Chon et al. also found a significantly improved local control for FSRT with a median of 35 Gy in 3–5 fractions compared to SRS with a median of 20 Gy: 1-year local control was 92.4% in the FSRT arm compared to 66.6% in the SRS arm (*p* = 0.028) (31). In addition, similarly to the work of Minniti et al. radiation necrosis was also markedly reduced in the FSRT arm with a radionecrosis rate of 0% in the FSRT arm compared to 39.8% for the SRS group (31). In the subgroup of large brain metastases, some negative comparative studies have also been published, which were mainly characterized by small patient numbers (32–34). However, we are unaware of any comparative investigations of FSRT and SRS that demonstrated significantly inferior results for the FSRT group. Lehrer et al. recently performed a large meta-analysis on the comparative efficacy and safety of FSRT and SRS in brain metastases >2 cm in diameter including 24 mainly non-comparative series on SRS and FSRT, respectively. While a significant difference in local control between the fractionated and single-session approach was not found, radiation necrosis was significantly increased in SRS studies (18.2% at 1 year) compared to 7.1% (*p* = 0.02) for FSRT series, that employed 3 × 9 Gy as their most common fractionation scheme (35).

Importantly however, our study was not limited to the more widely recognized FSRT-niche of larger brain metastases of >2–3 cm in diameter. Conversely, in the present investigation 0.065 cm^3^ (corresponding to a 5 mm diameter in a spherical lesion) was defined as the lower size limit exclusively to allow for accurate assessment of progression with the median tumor volume in the SRS group being 0.23 cm^3^ (median diameter 1.0 cm) and 1.42 cm^3^ in the FSRT group (median diameter 1.8 cm), respectively. In this group of smaller brain metastases, we observed significantly increased local control for FSRT compared to SRS (70.2 vs. 55.6% at 12 months), while the risk for radionecrosis was significantly decreased in the FSRT group (3.4 vs. 14.8% at 12 months). The effect on local control could be explained by a greater BED_12−LQC_ in the FSRT group, while the reduced risk for radiation necrosis could be attributed to a smaller BED_2−LQ_. The observed associations of fractionated treatment with increased local control and reduced risk for radionecrosis remained significant after accounting for known prognosticators in multivariate analysis including baseline tumor volume and histology among others.

We identified two other studies that retrospectively compared FSRT and SRS in smaller brain metastases. Kim et al. compared both treatment modalities in 98 patients and 109 brain metastases, respectively. In this series, the SRS group received a median of 20 Gy, while the FSRT group was treated with a median of 6 × 6 Gy. Median PTV volume was 2.2 cm^3^ in the SRS group, which would correspond to around 1.5 cm assuming a spherical configuration and 1 mm margin, while median PTV volume in the FSRT group was 5.0 cm^3^, corresponding to around 2 cm in tumor diameter. While local control was not different at 12 months (*p* = 0.31), toxicities were decreased from 17 to 5% in patients treated with FSRT (*p* = 0.05). Fokas et al. conducted a three-arm retrospective comparison of SRS according to RTOG 9005 and FSRT consisting of 7 × 5 Gy and 10 × 4 Gy in 214 patients, respectively. Median tumor volume in the SRS group was 0.87 cm^3^ (corresponding to a 1.2 cm diameter), 2.04 cm^3^ in the 7 × 5 Gy group (1.6 cm) and 5.93 cm^3^ in the 10 × 4 Gy arm (2.3 cm). Local control was not different between arms (*p* = 0.191), however interestingly radionecrosis occurred in 14% in the SRS group, 6% in the 7 × 5 Gy group and only in 2% in the 10 × 4 Gy arm (*p* = 0.01) (36).

To the best of our knowledge, the present study therefore is the first to report an advantage in local control for FSRT in smaller brain metastases. Potential explanations as to why the abovementioned series did not find a significant difference include the use of objective criteria based on longitudinal analysis for the definition of progression in the present study, which could have reduced misclassification. Assessment based on strict objective criteria could also have resulted in the overall local control being slightly lower in the present study when compared to other retrospective series. In the three large prospective randomized trials JROSG 99-1, EORTC 22952-26001 and the Alliance trial, local control rates of around 70% have consistently been shown for SRS alone at 12 months (4, 6, 7). The slightly lower local control rate observed for SRS in the present study can be attributed to melanoma brain metastases being the most common histology in the present series (43.7%), while non-small cell lung cancer constituted the most frequent primary in most other series including the three randomized controlled trials (4, 6, 7, 30). In addition, lesions <0.065 cm^3^ (~5 mm diameter) that might have achieved excellent control with SRS in the other series were excluded from the present analysis. Moreover, the larger study by Fokas et al. applied FSRT every other day, which may have reduced FSRT effectiveness because of repopulation or DNA damage repair and included patients (23%) that had received additional WBRT, which is known to enhance the local effectiveness of SRS (4, 6, 7, 36).

While improved local control for FSRT in smaller brain metastases is an unprecedented finding, it is perfectly consistent with current and past radiobiologic understanding. As early as 1993, Hall et al. argued against the then growing adoption of single-session radiosurgery for the treatment of small brain tumors, as brain metastases were seen as potentially hypoxic, typically early-reacting tissues characterized by a high α/β, whereas the surrounding brain was considered a classical late-reacting tissue with an α/β of around 2 (8). While additional effects of high fraction doses, like endothelial damage have been described and the use of the linear-quadratic model has been criticized for high single doses (37), the general view that brain metastases and surrounding normal brain tissue are characterized by fundamental radiobiologic differences holds true to this day (9). In 2011, Wiggenraad et al. published a systematic review of available SRS and FSRT studies for brain metastases in Radiotherapy and Oncology. When analyzing the relationship between radiotherapy dose and resulting local control in the literature, they found a clear dose-response relationship for an α/β ratio of 12 according to the linear-quadratic-cubic model (i.e., BED_12−LQC_). Of note, the linear-quadratic-cubic model further decreases the predicted effect of large fraction sizes compared to the linear-quadratic model (10).

Consequentially, as the fractionation effect seen in brain metastases is very low whereas that of the surrounding brain is very high, FSRT would be expected to provide an improved therapeutic ratio compared to SRS and therefore result in an improved ratio between local control and radionecrosis. It is interesting to note that local control indeed was highly significantly correlated with BED_12−LQC_ in the present analysis, while radionecrosis was significantly associated with BED_2−LQ_. Other effects of fractionated dose delivery could also be beneficial in brain metastases. In an *in silico* simulation study, Toma-Dasu et al. found that reoxygenation could profoundly improve tumor control in hypoxic brain metastases, if treatment was delivered fractionated rather than as single-session radiosurgery (38).

While the optimal fractionation scheme currently is unknown for FSRT in brain metastases, most studies favor 3–5 fractions (35). However, it is important to note, that if BED_12−LQC_ is predictive of local control and BED_2−LQ_ predicts radionecrosis, as suggested by current radiobiologic evidence, a higher ratio between the two would be achieved by a higher number of fractions. In this regard, it is worth emphasizing the relatively high median number of 10 fractions in the present study which could have contributed to the superior results in the FSRT group.

Only a small minority of patients had been treated with immune checkpoint inhibitors in the present study, which resulted in no detectable effect of checkpoint inhibitor treatment on radionecrosis risk in univariate and multivariate analysis. With the increasing use of checkpoint inhibitors in patients with brain metastases and reports of elevated risk of radionecrosis in patients undergoing PD-1 or CTLA-4 inhibitor treatment (39), a very interesting consideration is the potentially differing effect of concomitant immunotherapy on SRS and FSRT treatment. While large doses of radiation have been associated with a high effectiveness of eliciting local as well as systemic immune responses (40), decreased immunogenicity *via* DNA exonuclease Trex1 mediated DNA degradation has been described for radiation doses above 12–18 Gy (41). It is therefore an important research question, if—with concomitant immune checkpoint inhibitor treatment—FSRT in 2–3 fractions could be more immunogenic than single-session radiosurgery in terms of abscopal immune effects and systemic efficacy but also in regard to increased risk for radiation necrosis. Well-designed prospective trials with proper translational endpoints are important to further elucidate this interplay between fractionation and checkpoint inhibitor treatment in the future.

The overall prognosis of patients with brain metastases is undergoing profound improvement. While brain metastases used to confer a dismal prognosis, increasingly series of brain metastases with long-term survival are being reported (2, 3). Patients with long-term extracranial control would greatly benefit from improved intracranial control as well as reduced risk of radionecrosis and neurologic side effects to increase survival and maintain quality of life (42). The present study provides initial evidence that the improvements in therapeutic ratio observed for FSRT in larger brain metastases, might equally extend into the domain of smaller metastases, traditionally not considered for fractionated treatment.

In addition, the present study explores the use of volumetric criteria for the definition of progression in clinical trials for brain metastases. Volumetric criteria for progression were defined objectively and were derived from the current unidimensional RANO-BM criteria by following the overarching concept of the RANO-BM guideline to derive volumetric criteria from the established unidimensional ones using spherical geometry (14). The developed criteria were well-suited for the present analysis and resulted in comparable findings to conventional diameter measurements in the present study. Further research into volumetric criteria for brain metastases clinical trials is especially important and the developed criteria therefore have been implemented as secondary endpoint in an upcoming Phase III multicenter trial comparing SRS and FSRT in large brain metastases (NCT03697343).

### Limitations

The present analysis was to some extent limited by the number of included patients, which precluded certain subgroup analyses (e.g., for radioresistant brain metastases). Standardization in treatment benefited from the fact that all imaging and treatment was done at a single institution. However, due to the retrospective nature it cannot be excluded that treatment-related factors could have been influenced by hidden confounders.

Radionecrosis was defined volumetrically as transient increase in contrast enhancement followed by an objectively defined significant regression in the absence of treatment (local or systemic) and not based on advanced MRI techniques or PET, which may be regarded as limitation. However, we believe that this approach might not have been inferior, as the abovementioned modalities also fail to provide a definitive diagnosis and the retrospective design contributed to the fact that additional imaging follow-up was available, which has an accepted role in distinguishing radionecrosis from progression (43). Moreover, reduced risk of radionecrosis was also consistently observed in the subgroup with histologic confirmation.

## Conclusion

Current radiobiologic understanding predicts that fractionation should be able to increase the therapeutic ratio in SRT for brain metastases. In this volumetric study using objective volume-based progression criteria derived from current unidimensional RANO-BM recommendations, we found a significant increase in local control and a significantly reduced risk for radionecrosis in metastases treated with FSRT compared to SRS. Consistent with radiobiologic models, we found a strong association of local control probability with BED_12−LQC_ and of radionecrosis risk with BED_2−LQ_. The present study provides initial evidence that the improvements in therapeutic ratio expected for FSRT in larger brain metastases, might equally extend into the domain of smaller metastases, traditionally less considered for fractionated treatment. FSRT might constitute an important tool to further increase local control and reduce radionecrosis risk in stereotactic radiotherapy for brain metastases, that should be assessed in randomized intervention trials.

## Data Availability Statement

The raw data supporting the conclusions of this article will be made available by the authors, without undue reservation.

## Ethics Statement

Ethical review and approval was not required for the study on human participants in accordance with the local legislation and institutional requirements. The patients/participants provided their written informed consent to participate in this study.

## Author Contributions

FP, TW, DO, MS, SL, LD, and RF conceptualized the manuscript. FP, DO, MS, and TW investigated the findings. FP, DO, JR, and HS performed the analysis. MS, VM, SS, CB, SL, BF, LD, RF, and FP provided the resources. FP, TW, MS, JR, HS, VM, CS, IF, CB, SL, BF, LD, SS, and RF performed the writing. IF, CB, BF, LD, SS, RF, and FP supervised the findings. All authors contributed to the article and approved the submitted version.

## Conflict of Interest

The authors declare that the research was conducted in the absence of any commercial or financial relationships that could be construed as a potential conflict of interest.

## References

[1] Tabouret E Chinot O Metellus P Tallet A Viens P Goncalves A. Recent trends in epidemiology of brain metastases: an overview. Anticancer Res. (2012) 32:4655–62. 23155227

[2] Johung KL Yeh N Desai NB Williams TM Lautenschlaeger T Arvold ND. Extended survival and prognostic factors for patients with ALK-rearranged non-small-cell lung cancer and brain metastasis. J Clin Oncol. (2016) 34:123–9. 10.1200/JCO.2015.62.0138 26438117 PMC5070549

[3] Gaudy-Marqueste C Dussouil AS Carron R Troin L Malissen N Loundou A. Survival of melanoma patients treated with targeted therapy and immunotherapy after systematic upfront control of brain metastases by radiosurgery. Eur J Cancer. (2017) 84:44–54. 10.1016/j.ejca.2017.07.017 28783540

[4] Brown PD Jaeckle K Ballman KV Farace E Cerhan JH Anderson SK. Effect of radiosurgery alone vs radiosurgery with whole brain radiation therapy on cognitive function in patients with 1 to 3 brain metastases: a randomized clinical trial. JAMA. (2016) 316:401–9. 10.1001/jama.2016.9839 27458945 PMC5313044

[5] Barbour AB Jacobs CD Williamson H Floyd SR Suneja G Torok JA. Radiation therapy practice patterns for brain metastases in the united states in the stereotactic radiosurgery era. Adv Radiat Oncol. (2020) 5:43–52. 10.1016/j.adro.2019.07.012 32051889 PMC7004940

[6] Aoyama H Shirato H Tago M Nakagawa K Toyoda T Hatano K. Stereotactic radiosurgery plus whole-brain radiation therapy vs stereotactic radiosurgery alone for treatment of brain metastases: a randomized controlled trial. JAMA. (2006) 295:2483–91. 10.1001/jama.295.21.2483 16757720

[7] Kocher M Soffietti R Abacioglu U Villa S Fauchon F Baumert BG Adjuvant whole-brain radiotherapy versus observation after radiosurgery or surgical resection of one to three cerebral metastases: results of the EORTC 22952-26001 study. J Clin Oncol. (2011) 29:134–41. 10.1200/JCO.2010.30.1655 21041710 PMC3058272

[8] Hall EJ Brenner DJ. The radiobiology of radiosurgery: rationale for different treatment regimes for AVMs and malignancies. Int J Radiat Oncol Biol Phys. (1993) 25:381–5. 10.1016/0360-3016(93)90367-5 8420891

[9] Kondziolka D Shin SM Brunswick A Kim I Silverman JS. The biology of radiosurgery and its clinical applications for brain tumors. Neuro Oncol. (2015) 17:29–44. 10.1093/neuonc/nou284 25267803 PMC4483054

[10] Wiggenraad R Verbeek-de Kanter A Kal HB Taphoorn M Vissers T Struikmans H. Dose-effect relation in stereotactic radiotherapy for brain metastases. A systematic review. Radiother Oncol. (2011) 98:292–7. 10.1016/j.radonc.2011.01.011 21316787

[11] Fuks Z Kolesnick R. Engaging the vascular component of the tumor response. Cancer Cell. (2005) 8:89–91. 10.1016/j.ccr.2005.07.014 16098459

[12] Thiagarajan A Yamada Y. Radiobiology and radiotherapy of brain metastases. Clin Exp Metast. (2017) 34:411–9. 10.1007/s10585-017-9865-7 29139010

[13] Soffietti R Abacioglu U Baumert B Combs SE Kinhult S Kros JM. Diagnosis and treatment of brain metastases from solid tumors: guidelines from the European association of neuro-oncology (EANO). Neuro Oncol. (2017) 19:162–74. 10.1093/neuonc/now241 28391295 PMC5620494

[14] Lin NU Lee EQ Aoyama H Barani IJ Barboriak DP Baumert BG. Response assessment criteria for brain metastases: proposal from the RANO group. Lancet Oncol. (2015) 16:e270–8. 10.1016/S1470-2045(15)70057-4 26065612

[15] Blonigen BJ Steinmetz RD Levin L Lamba MA Warnick RE Breneman JC. Irradiated volume as a predictor of brain radionecrosis after linear accelerator stereotactic radiosurgery. Int J Radiat Oncol Biol Phys. (2010) 77:996–1001. 10.1016/j.ijrobp.2009.06.006 19783374

[16] Koca S Distel L Lubgan D Weissmann T Lambrecht U Lang-Welzenbach M. Time course of pain response and toxicity after whole-nerve-encompassing LINAC-based stereotactic radiosurgery for trigeminal neuralgia-a prospective observational study. Strahlenther Onkol. (2019) 195:745–55. 10.1007/s00066-019-01450-9 30877350

[17] Putz F Muller J Wimmer C Goerig N Knippen S Iro H. Stereotactic radiotherapy of vestibular schwannoma: hearing preservation, vestibular function, and local control following primary and salvage radiotherapy. Strahlenther Onkol. (2017) 193:200–12. 10.1007/s00066-016-1086-5 27928625

[18] Joiner M Quantifying cell kill and survival. In: Joiner M editor. Basic Clinical Radiobiology. London, England: Hodder Arnold (2009). p. 52. 10.1201/b13224-5

[19] Fedorov A Beichel R Kalpathy-Cramer J Finet J Fillion-Robin JC Pujol S. 3D Slicer as an image computing platform for the quantitative imaging network. Mag Resonance Imaging. (2012) 30:1323–41. 10.1016/j.mri.2012.05.001 22770690 PMC3466397

[20] Pinter C Lasso A Wang A Jaffray D Fichtinger G. SlicerRT: radiation therapy research toolkit for 3D slicer. Med Phys. (2012) 39:6332–8. 10.1118/1.4754659 23039669

[21] Weissmann T Lettmaier S Roesch J Mengling V Bert C Iro H. Paragangliomas of the head and neck: local control and functional outcome following fractionated stereotactic radiotherapy. Am J Clin Oncol. (2019) 42:818–23. 10.1097/COC.0000000000000614 31592806

[22] Zhu L Kolesov I Gao Y Kikinis R Tannenbaum A An effective interactive medical image segmentation method using Fast GrowCut. In: Int Conf Med Image Comput Comput Assist Interv Workshop on Interactive Methods. Boston, MA (2014).

[23] Zhao B Tan Y Tsai WY Qi J Xie C Lu L. Reproducibility of radiomics for deciphering tumor phenotype with imaging. Sci Rep. (2016) 6:23428. 10.1038/srep23428 27009765 PMC4806325

[24] Balagurunathan Y Kumar V Gu Y Kim J Wang H Liu Y. Test-retest reproducibility analysis of lung CT image features. J Digit Imaging. (2014) 27:805–23. 10.1007/s10278-014-9716-x 24990346 PMC4391075

[25] Odland A Server A Saxhaug C Breivik B Groote R Vardal J. Volumetric glioma quantification: comparison of manual and semi-automatic tumor segmentation for the quantification of tumor growth. Acta Radiol. (2015) 56:1396–403. 10.1177/0284185114554822 25338837

[26] Shaw E Scott C Souhami L Dinapoli R Kline R Loeffler J. Single dose radiosurgical treatment of recurrent previously irradiated primary brain tumors and brain metastases: final report of RTOG protocol 90-05. Int J Radiat Oncol Biol Phys. (2000) 47:291–8. 10.1016/S0360-3016(99)00507-6 10802351

[27] Tsao MN Rades D Wirth A Lo SS Danielson BL Gaspar LE. Radiotherapeutic and surgical management for newly diagnosed brain metastasis(es): an American society for radiation oncology evidence-based guideline. Pract Radiat Oncol. (2012) 2:210–25. 10.1016/j.prro.2011.12.004 25925626 PMC3808749

[28] National Comprehensive Cancer Network Central Nervous System Cancers (Version 1.2020). (2020). Available online from: https://www.nccn.org/professionals/physician_gls/pdf/cns.pdf (accessed March 01, 2020).

[29] Zindler JD Schiffelers J Lambin P Hoffmann AL. Improved effectiveness of stereotactic radiosurgery in large brain metastases by individualized isotoxic dose prescription: an *in silico* study. Strahlenther Onkol. (2018) 194:560–9. 10.1007/s00066-018-1262-x 29349605 PMC5959984

[30] Minniti G Scaringi C Paolini S Lanzetta G Romano A Cicone F Single-fraction versus multifraction (3 x 9 Gy) stereotactic radiosurgery for large (>2 cm) brain metastases: a comparative analysis of local control and risk of radiation-induced brain necrosis. Int J Radiat Oncol Biol Phys. (2016) 95:1142–8. 10.1016/j.ijrobp.2016.03.013 27209508

[31] Chon H Yoon K Lee D Kwon DH Cho YH. Single-fraction versus hypofractionated stereotactic radiosurgery for medium-sized brain metastases of 2.5 to 3 cm. J Neuro Oncol. (2019) 145:49–56. 10.1007/s11060-019-03265-1 31420793

[32] Donovan EK Parpia S Greenspoon JN. Incidence of radionecrosis in single-fraction radiosurgery compared with fractionated radiotherapy in the treatment of brain metastasis. Curr Oncol. (2019) 26:e328–e33. 10.3747/co.26.4749 31285676 PMC6588068

[33] Park K Kim JW Chung HT Paek SH Kim DG. Single-session versus multisession gamma knife radiosurgery for large brain metastases from non-small cell lung cancer: a retrospective analysis. Stereotact Func Neurosurg. (2019) 97:94–100. 10.1159/000496154 31117101

[34] Feuvret L Vinchon S Martin V Lamproglou I Halley A Calugaru V. Stereotactic radiotherapy for large solitary brain metastases. Cancer Radiother. (2014) 18:97–106. 10.1016/j.canrad.2013.12.003 24439342

[35] Lehrer EJ Peterson JL Zaorsky NG Brown PD Sahgal A Chiang VL. Single versus multifraction stereotactic radiosurgery for large brain metastases: an international meta-analysis of 24 trials. Int J Radiat Oncol Biol Phys. (2019) 103:618–30. 10.1016/j.ijrobp.2018.10.038 30395902

[36] Fokas E Henzel M Surber G Kleinert G Hamm K Engenhart-Cabillic R. Stereotactic radiosurgery and fractionated stereotactic radiotherapy: comparison of efficacy and toxicity in 260 patients with brain metastases. J Neuro Oncol. (2012) 109:91–8. 10.1007/s11060-012-0868-6 22528795

[37] Brown JM Carlson DJ Brenner DJ. The tumor radiobiology of SRS and SBRT: are more than the 5 Rs involved? Int J Radiat Oncol Biol Phys. (2014) 88:254–62. 10.1016/j.ijrobp.2013.07.022 24411596 PMC3893711

[38] Toma-Dasu I Sandstrom H Barsoum P Dasu A To fractionate or not to fractionate? That is the question for the radiosurgery of hypoxic tumors. J Neurosurg. (2014) 121:110–5. 10.3171/2014.8.GKS141461 25434944

[39] Martin AM Cagney DN Catalano PJ Alexander BM Redig AJ Schoenfeld JD. Immunotherapy and symptomatic radiation necrosis in patients with brain metastases treated with stereotactic radiation. JAMA Oncol. (2018) 4:1123–4. 10.1001/jamaoncol.2017.3993 29327059 PMC5885198

[40] Herskind C Wenz F Giordano FA. Immunotherapy combined with large fractions of radiotherapy: stereotactic radiosurgery for brain metastases-implications for intraoperative radiotherapy after resection. Front Oncol. (2017) 7:147. 10.3389/fonc.2017.00147 28791250 PMC5522878

[41] Vanpouille-Box C Alard A Aryankalayil MJ Sarfraz Y Diamond JM Schneider RJ. DNA exonuclease Trex1 regulates radiotherapy-induced tumour immunogenicity. Nat Commun. (2017) 8:15618. 10.1038/ncomms15618 28598415 PMC5472757

[42] Steinmann D Vordermark D Gerstenberg W Aschoff R Gharbi N Muller A. Quality of life in patients with limited (1-3) brain metastases undergoing stereotactic or whole brain radiotherapy : a prospective study of the DEGRO QoL working group. Strahlenther Onkol. (2020) 196:48–57. 10.1007/s00066-019-01506-w 31418046

[43] Chao ST Ahluwalia MS Barnett GH Stevens GH Murphy ES Stockham AL. Challenges with the diagnosis and treatment of cerebral radiation necrosis. Int J Radiat Oncol Biol Phys. (2013) 87:449–57. 10.1016/j.ijrobp.2013.05.015 23790775

